# Using expression quantitative trait loci data and graph-embedded neural networks to uncover genotype–phenotype interactions

**DOI:** 10.3389/fgene.2022.921775

**Published:** 2022-08-15

**Authors:** Xinpeng Guo, Jinyu Han, Yafei Song, Zhilei Yin, Shuaichen Liu, Xuequn Shang

**Affiliations:** ^1^ School of Computer Science and Engineering, Northwestern Polytechnical University, Xi’an, China; ^2^ School of Air and Missile Defense, Air Force Engineering University, Xi’an, China; ^3^ School of Economics and Management, Chang ‘an University, Xi’an, China; ^4^ School of Marine Science and Technology, Northwestern Polytechnical University, Xi’an, China

**Keywords:** eQTL, expression quantitative trait loci, graph-embedded deep neural network, genotype-phenotype, SNP, gene

## Abstract

**Motivation:** A central goal of current biology is to establish a complete functional link between the genotype and phenotype, known as the so-called genotype**–**phenotype map. With the continuous development of high-throughput technology and the decline in sequencing costs, multi-omics analysis has become more widely employed. While this gives us new opportunities to uncover the correlation mechanisms between single-nucleotide polymorphism (SNP), genes, and phenotypes, multi-omics still faces certain challenges, specifically: 1) When the sample size is large enough, the number of omics types is often not large enough to meet the requirements of multi-omics analysis; 2) each omics’ internal correlations are often unclear, such as the correlation between genes in genomics; 3) when analyzing a large number of traits (*p*), the sample size (*n*) is often smaller than *p*, *n << p,* hindering the application of machine learning methods in the classification of disease outcomes.

**Results:** To solve these issues with multi-omics and build a robust classification model, we propose a graph-embedded deep neural network (G-EDNN) based on expression quantitative trait loci (eQTL) data, which achieves sparse connectivity between network layers to prevent overfitting. The correlation within each omics is also considered such that the model more closely resembles biological reality. To verify the capabilities of this method, we conducted experimental analysis using the GSE28127 and GSE95496 data sets from the Gene Expression Omnibus (GEO) database, tested various neural network architectures, and used prior data for feature selection and graph embedding. Results show that the proposed method could achieve a high classification accuracy and easy-to-interpret feature selection. This method represents an extended application of genotype–phenotype association analysis in deep learning networks.

## Introduction

A central goal of current biology is to establish a complete functional link between the genotype and phenotype, known as the so-called genotype-phenotype map (Romanowska and Joshi, 2019). Studying the relationship between the genotype and phenotype can clarify the process of genetic variation (Lunenburg et al., 2021; Tsuji et al., 2021). Genome-wide association studies (GWAS) between common genotypes and phenotypes are an effective way to reveal the link between an individual’s genetic background and a specific disease or trait. The principle is to find all the genetic variants on the genome (most often single-nucleotide polymorphisms, or SNPs) and analyze the correlation between genetic variants and phenotypes (Slaten et al., 2020). Over the past decade, numerous GWASs have identified many genetic variants associated with complex human diseases or other traits (Lv et al., 2017; Wong et al., 2017; Shashkova et al., 2021). Their findings indicate novel variant-trait associations (Meyer and PhenotypeSimulator, 2018; Nussinov et al., 2019) and provide a variety of new methods for analyzing complex traits (Fortune et al., 2019; Sealfon et al., 2020; Denault et al., 2021), enriching a multitude of clinical applications (Kim et al., 2016; Maier et al., 2018). However, according to the principles of GWASs, while SNPs for thousands of complex diseases and traits have been discovered, single omics studies can only illuminate a limited number of biological mechanisms, and the further functional implications and mechanisms of the relevant loci are largely unclear.

In recent years, a growing number of studies have attempted to link clinical outcomes (e.g., for cancer and other diseases) to gene expression and other types of omics data (Athreya and Lazaridis, 2021; Hulot et al., 2021; Jendoubi, 2021) to create multi-omics data for analysis (Dimitrakopoulos et al., 2018; Rao et al., 2018; Zhao et al., 2018; Duan et al., 2021; Guan et al., 2021), making such studies more biologically realistic. For example, only those SNPs that affect the phenotype can be known through GWAS data, but if the gene expression data is added to the SNP and phenotype omics data, the SNP-gene-phenotype pathway relationship can be solved by using the multi-omics data method. So that makes the biological significance even clearer. However, multi-omics data in genotype–phenotype association analysis, especially in some novel disease data sets, are generally vulnerable to the following two situations (Ritchie et al., 2015; Lin and Lane, 2017; Picard et al., 2021). First, with the increase of omics data types, multi-omics data on the same sample are not easy to obtain, resulting in small multi-omics sample sizes. For example, we made statistics on the data in the whole GEO database and found that when there were four omics data types, there were only two groups of data with 100–300 sample sizes. When there were three omics data types, there were two groups with 300–400 sample sizes, five groups with 200–300 sample sizes, and four groups with 100–200 sample sizes (up to 30 June 2022). According to NETAM method (Lee et al., 2016), when the sample size is N > 200, its method will have certain effect, and when the sample size is equal to 800, the performance will reach the best. The problem of multi-omics data analysis in such small sample cases has been investigated in another article of mine (Guo et al., 2021). Second, if the sample size is large enough, the number of omics types is often small. We find in the GEO database sample size can reach more than 1,000, but its omics data contains only genotype and phenotype two omics type, and falling short of the requirements for multi-omics applications, how to make use of such data, combined with the advantages of multiple omics data analysis, understanding the relationship between genotype and phenotypic pathway is a problem to be solved in this paper.

Correlation between omics is often used to achieve multi-omics analysis (as when studying the effect of SNPs on phenotypes), and a common method is the introduction of gene expression through embedding eQTL data, which can lead to better classification performance (Zhu et al., 2016; Shan et al., 2019; Wu et al., 2019; Gerring et al., 2021). However, owing to the massive number of features of SNPs, dimensionality reduction methods should be applied before eQTL use, such as the commonly used principal component analysis (PCA) or Isomap in manifold learning. Still, such unsupervised dimensionality reduction methods cannot obtain the pathway relationships between SNPs, gene effects, and disease. To reduce the amount of input features, feature filtering methods can be used in addition to dimensionality reduction methods. While the neural network model is a multilayer network architecture, the number of nodes in the middle layer is usually fewer than the number of input values. Intermediate layers with a smaller cardinality (compared to the number of inputs) can be used as a reduced dimensional representation of the input data. A supervised method of feature filtering proposed by Lin et al. builds upon a neural network to generate a low-dimensional representation of single-cell RNA-sequencing (scRNA-seq) data (Lin et al., 2017). By combining neural networks with protein–protein interaction (PPI) networks and using the hidden layer of the neural network to generate a low-dimensional representation of scRNA-seq data, this method can achieve better performance than most existing unsupervised models, effectively reducing the dimensionality of SNPs. With genes as our intermediate layer, we use eQTL data to establish the association between SNPs and genes, allowing the network to capture important biological knowledge present in the data, obtain the SNP–gene–phenotype association, and filter the number of SNP to reduce dimensionality. In this way, other prior knowledge is utilized to achieve the analysis effect of multiple sets of data without multiple sets of data.

Additionally, diseases do not arise from merely a few genes, but rather from interactions between genes with impacts on the disease. In other words, functionally related genes are more likely to be interdependent and impact biological outcomes in a synergistic manner. For example, our team created the IPMM (Integration Pathways and Motif Model) by combining genetic association information such as pathways and motifs with multi-omics data to study cancer subtype classification. Our results show that the clustering effect was improved to varying degrees in each method after incorporating the intergenic associations (Guo et al., 2020). Therefore, implications of the biological association between genes should be considered when introducing genetic information into multi-omics. Kong et al. combined gene networks with deep feedforward networks (DFNs), which are useful in both classification performance and learning the structure of trait spaces (Kong and Yu, 2018). Zhao et al. proposed a new machine-learning-based framework, GCN-DTI, for identification of new drug–target interactions. The model first uses a graph convolutional network (GCN) to learn the features of each drug–protein pair (DPP). Then, using the feature representation as input, the model uses a deep neural network (DNN) to predict the final label. Their framework is largely superior to some of the most advanced methods (Zhao et al., 2021). The above method not only considers the correlation between genes, but also effectively combines gene networks with deep learning networks.

In summary, we propose a graph-embedded deep neural network (G-EDNN) based on eQTL data, which implements sparse connectivity and feature filtering between network layers to prevent overfitting and reduce dimensionality, respectively. Our model also embeds the gene network into the broader neural network to integrate intra-omics correlation and better reflect the biological reality. By effectively filtering the eQTL and gene correlation layer relationships, we resolve the issue of the sample size (*n*) being smaller than number of features *p*, *n << p*, which has historically hindered the application of machine learning methods in the classification of disease outcomes. We performed experimental analysis using the GSE28127 and GSE95496 data sets from the GEO database, tested various neural network architectures, and used prior data for feature selection and graph embedding. The results show that the proposed method could achieve high classification accuracy and easy-to-interpret feature selection. The method extends the application of deep learning networks to genotype–phenotype association analysis.

The main contributions of this paper include:

1. Increasing interpretability of DNNs by integrating SNPs with genes and incorporating intergenic correlations.
2. Simultaneously analyzing inter-omics and intra-omics relationships, which is more in line with actual biological mechanisms.
3. Achieving the dual purposes of feature filtering and dimensionality reduction using neural networks.
4. Using GWAS data and other prior data to attain multi-omics analysis, which clarifies pathway relationships between SNPs, genes, and disease.

## Methods

### DNNs

DNNs are the most common deep learning architecture. An *n*-layer network can be represented as

$$X_{1}=\delta \left(X_{0}W_{0}+b_{0}\right)$$

$$X_{i}=\delta \left(X_{i-1}W_{i-1}+b_{i-1}\right)$$

$$X_{n}=\delta \left(X_{n-1}W_{n-1}+b_{n-1}\right)$$

$$P\left(Y|X_{0},\theta \right)=f\left(X_{\text{n}}W_{n}+b_{n}\right)$$

wherein $X_{0}$ is an input matrix of *n* sample sizes; *P* is features; $W_{0}$ , $b_{0}$ is the initialization weight matrix and offset, respectively; Y is the n-dimensional sample label; θ represents all parameters of the model; and $X_{i}$ , $W_{i}$ , $b_{i}\left(i=1,...,n\right)$ represents each neuron layer, weight matrix, and offset, respectively. δ(·) is the activation function, such as a Sigmoid function, Tanh function, or ReLU function. f(·) denotes the conversion of the values of the output layers into probabilistic predictions using the softmax function. Algorithms such as stochastic gradient descent (SGD) are used to optimize the loss function, changing the parameters to minimize cross-entropy loss (Courville, 2016).

$$C=-\frac{1}{n}\sum_{i=1}^{n}Y_{i}\ln\left(f\left(X_{i}W_{i}+b_{i}\right)\right)+\left(1-Y_{i}\right)\ln\left(1-f\left(X_{i}W_{i}+b_{i}\right)\right)$$

### G-EDNN based on eQTL data

**FIGURE 1:**
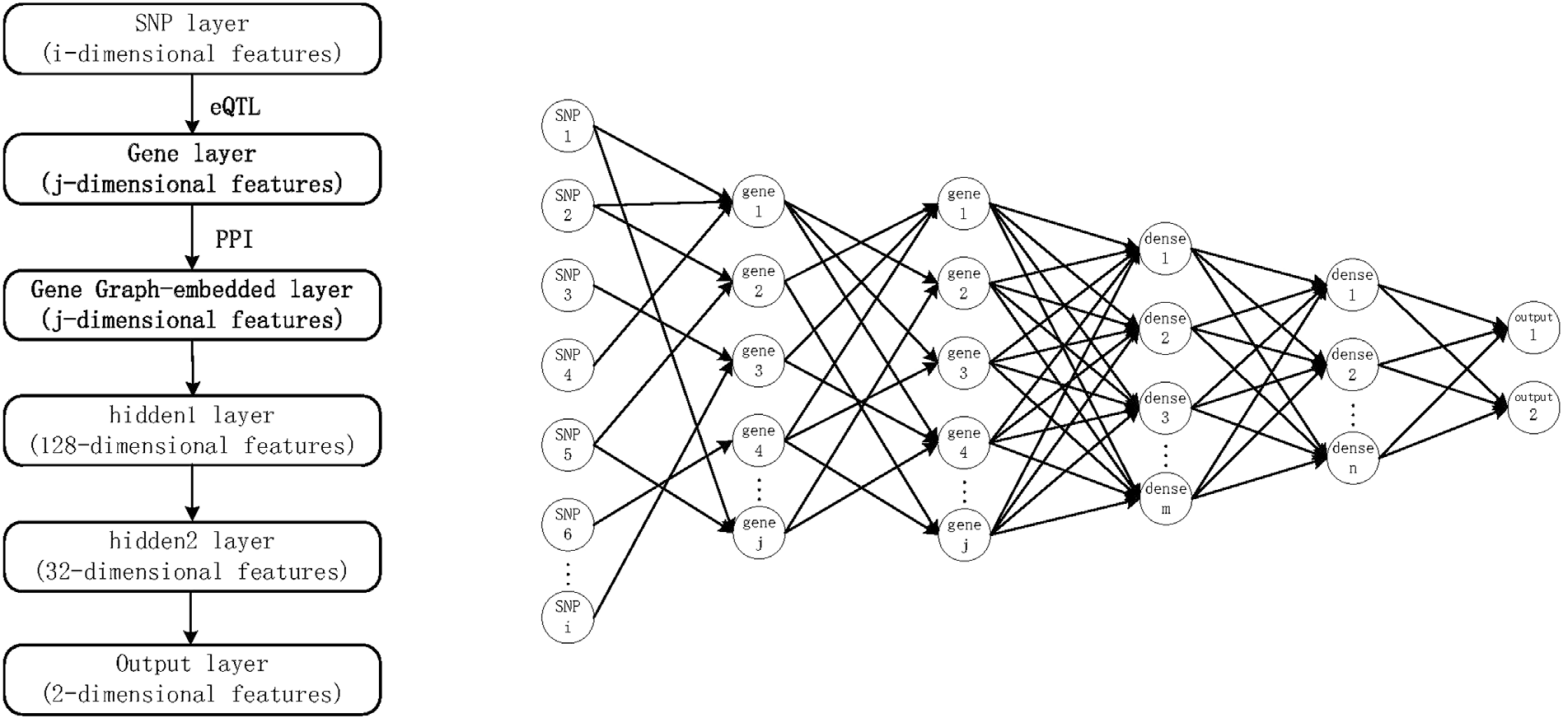
G-EDNN model diagram based on expression quantitative trait loci data. The intergenic correlation network is embedded into a DNN to form the final G-EDNN model.

Using the above DNN approach, SNPs can be used as input and the phenotype as output to build a deep learning approach. To incorporate known prior knowledge into DNNs, we propose the G-EDNN model based on eQTL data. This model makes two major prerequisite assumptions: 1) SNPs influence the phenotype through genes; and 2) the influence of the genotype on phenotype does not rely on an individual gene, but on how intergenic relationships cumulatively impact the phenotype. For the first prerequisite, it has been demonstrated in literature (Lee et al., 2016) that many SNPs influence the phenotype through genes. Thus, we added the intermediate gene network layer, which better corresponds with biological reality and additionally allows for sparse connectivity between layers using eQTL data. Further, in multilayer neural network model structures, usually, the number of downstream nodes is fewer than the number of input values, which can be leveraged to represent a dimensionality-reduced version of the input data. Moreover, as genes have interactive correlative relationships, which do not meet the characteristics of independent and homogeneous neuron distribution of a hidden layer, an intergenic correlation network should be integrated directly into the neural network. This gives rise to the second prerequisite assumption, also supported in the literature (Kong and Yu, 2018), that the model has higher classification accuracy and better feature selection when the correlations between genes are incorporated in the neural network, as shown in Figure 1.

Sparse connectivity is involved in both SNP–gene correlation and intergenic correlations. The correlations can be represented by adjacency matrices. Let A and A′ denote the adjacency matrix between the SNP–gene and gene–gene correlation, respectively. The relationship between SNP and gene came from eQTL data, and the association data between genes came from PPI data. Then,

$$A_{ij}=\{\begin{array}{cc} 1\text{,} & if\;SNP\;and\;gene\;are\;connected\\ \text{0}\text{,} & otherwise \end{array}A_{ij}^{\prime }=\{\begin{array}{cc} 1\text{,} & if\;gene\;and\;gene\;are\;connected\\ \text{0}\text{,} & otherwise \end{array}$$

where i and j represent the matrix coordinates; and A′ is the symmetrical matrix such that $A_{jj}^{\prime }=1$ , indicating that the upper and lower layers of the gene network interconnect.

By incorporating two adjacency matrices ( A and A′ ) into the DNN network, the original equation becomes

$$X_{1}=\delta \left(X_{0}(W_{0}⊙A)+b_{0}\right)$$

$$X_{2}=\delta \left(X_{1}(W_{1}⊙A\prime )+b_{1}\right)$$

$$X_{3}=\delta \left(X_{2}W_{2}+b_{2}\right)$$

$$X_{k}=\delta \left(X_{k-1}W_{k-1}+b_{k-1}\right)$$

$$P\left(Y|X_{0},\theta \right)=f\left(X_{\text{n}}W_{n}+b_{n}\right)$$

where the operator ⊙ denotes the Hadamard product (element-wise product), k = 3, …, n represents the number of hidden neuron layer. Thus, the connections between the first three layers of the feedforward network achieve feature filtering through the dot-product adjacency matrix. SNPs are selected using genomics, and the intergene correlations are also considered. The SNP layer keeps those SNPs related to genes, and the gene layer keeps those genes related to both the SNPs and another gene. Because feature selection is based on the predictive model, and not reliant upon only the feature’s mathematical characteristics, the selected features may help elucidate underlying biological mechanisms of disease.

### Detailed model settings

The G-EDNN’s detailed settings include selecting the activation function, the optimizer, the overfit prevention strategy, the learning rate, the number of layers, the number of hidden layer nodes, and the batch value, among other settings. The selection of these values is not the focus of our work. The hyperparameter tuning process is guided by the area under the validation curve (AUC) of the receiver operating characteristics (ROC) curve. In other words, we will select the best candidate hyperparameter based on the AUC validation curve score.

For training the G-EDNN, we used the ReLU activation function (Nair and Hinton, 2010), which has advantages over sigmoid and tanh functions because it avoids the vanishing gradients problem when using SGD (Kolen and Kremer, 2001). For the optimizer, we chose the Adam optimization algorithm (Kingma and Ba, 2014), which is an extension of the most widely used SGD algorithm in deep learning. In addition, we used a small-batch training strategy where the optimizer trains a small number of samples randomly in each iteration. Too large a batch size will reduce the network’s accuracy because it will reduce the stochasticity of gradient descent. All else being equal, the larger the batch size, the more epochs that need to be trained to achieve the same accuracy, whereas with a smaller batch size, more weight updates can be performed per epoch. There are two advantages here: First, the epoch can quickly identify local minima; and second, it has better generalization capacities. Thus, we used smaller batch sizes, such as 16, 8, or even 1. The learning rate setting generally does not affect classification performance but may lead to inconsistent convergence speeds. A relatively large learning rate will speed up convergence but may also risk skipping the optimal value. We set the candidate rates to 0.05, 0.01, 0.005, and 0.001, and the combination of a learning rate of 0.001 and batch size of eight was found to be the best choice.

To avoid the overfitting phenomenon, we used the dropout strategy. The strategy aims to randomly remove neurons in the network (except the output layer) according to a certain probability during the training process. The use of dropout prevents the parameters from being overly dependent on the training data and increases the ability of the parameters to generalize to the data set. After testing several values of 0.5, 0.6, 0.7, 0.8, and 0.9, we settled on a dropout parameter value of 0.9. We simultaneously used regularization to mitigate overfitting. Regularization introduces a model complexity metric into the loss function to weaken the noise of training data using a weight matrix ( $W_{i}$ ) with the formula

$$Loss=loss\left(Y,f\left(X_{\text{n}}W_{n}+b_{n}\right)\right)+\alpha *\sum_{\text{i}=0}^{m}loss\left(W_{i}\right)$$

$loss(Y,f\left(X_{\text{n}}W_{n}+b_{n}\right))$ . Is the loss function of all parameters in the model, such as cross entropy. The hyperparameter α defines the proportion of $\sum_{\text{i}=0}^{m}loss\left(W_{i}\right)$ over total loss, which is the weight of the regularization, and *m* is the number of layers of the neural network.

In addition, the neural network has two important hyperparameters that control the network topology’s number of layers and number of nodes per layer. The values of these parameters must be specified when configuring the network. The number of hidden layers cannot be too high, as we are dealing with relatively small samples. Further, too shallow of a neural network is detrimental to our complex classification task. With the above considerations, we tested three to five hidden layers (not including the gene layer). For the hidden neurons, we followed the convention in deep learning to set the numbers as powers from the input layer to the output layer, decreasing in magnitude. In fact, the number of hidden nodes usually has a rather small impact on the performance of a neural network compared to other factors. In many cases, increasing the number of hidden units needed simply slows down the training speed. Compared with ordinary neural networks, the G-EDNN model has certain unique features. The number of neurons in the gene layer depends on the correlation with the SNP layer, as well as within its own layer. For the other hidden layers, we tested numbers from 256, 128, 64, 32 and 16. When the model included a gene hidden layer, then two other hidden layers were selected, with 128 and 16 nodes per hidden layer, respectively. If the model did not include a hidden gene layer, then three hidden layers were selected, with 256, 64, and 16 nodes per layer, respectively.

## Results

### Data sources and preprocessing

To validate this method, we used two data sets from the Gene Expression Omnibus (GEO) database (Edgar ett al., 2002). GSE28127 (Lamb et al., 2011) is a study of hepatocellular carcinoma patient data, which uses the Illumina HumanHap650Yv3 Genotyping BeadChip (HumanHap650Yv3_A) chip to analyze DNA and expression variants in 217 tumor cancer patients and 184 non-tumor cancer patients. GSE95496 data (Vujkovic et al., 2017) were used to conduct SNP analysis using Affymetrix 6.0 SNP arrays on 254 pairs of acute myeloid leukemia samples (remission or diagnostic).

PPI network data were derived from Protein InteraCtion KnowLedgebasE (PICKLE) (Gioutlakis et al., 2017). This database is a meta-database of human protein interactions, integrating publicly available PPI interaction databases via genetic information ontology.

The eQTL data originated from GTEx Analysis V7 (dbGaP Accession phs000424. v7. p2) (Lonsdale et al., 2013). To preserve the accuracy of data prediction and combat tissue idiosyncrasies, we selected eQTL data that corresponded with our data. For example, we selected hepatic eQTL data for the first data set, and blood eQTL data for the second data set.

Data preprocessing mainly involved unifying SNP and gene naming in various types of data and removing data with missing values of more than 20% in SNP data. If less than 20% of data was missing values, then we filled missing data with the highest frequency value or with the average value and only retained SNP s with a minor allele frequency (MAF) greater than 0.05.

### Prediction analysis

The method in this paper is based on neural networks, integrating prior information such as eQTL in the first layer, and considering intergenic correlations in the hidden layer. We selected the following additional methods for comparative experiments:

1) General DNN. This method uses traditional deep learning networks, various parameter settings, and activation function selection. The process of feature selection through prior information such as eQTL data is removed from the first layer, and gene-related information is not considered in the hidden layer.
2) E-DNN. Based on general DNN, SNPs are feature-filtered by a first layer of eQTL data. This method evolved from the feature selection method proposed by Chieh Lin et al. (Lin et al., 2017), combined with SNP data characteristics. This method does not consider intergenic correlations.
3) Graph-embedded deep feedforward network (GEDFN). Kong et al. (Kong and Yu, 2018) proposed the GEDFN method, which considers intergenic correlations and resolves sample sizes being much smaller than the number of features by integrating the gene network directly into the classifier. In the GEDFN method, the SNP and gene layers are fully connected, and only gene network information is incorporated into the hidden layer for feature screening.

**FIGURE 2:**
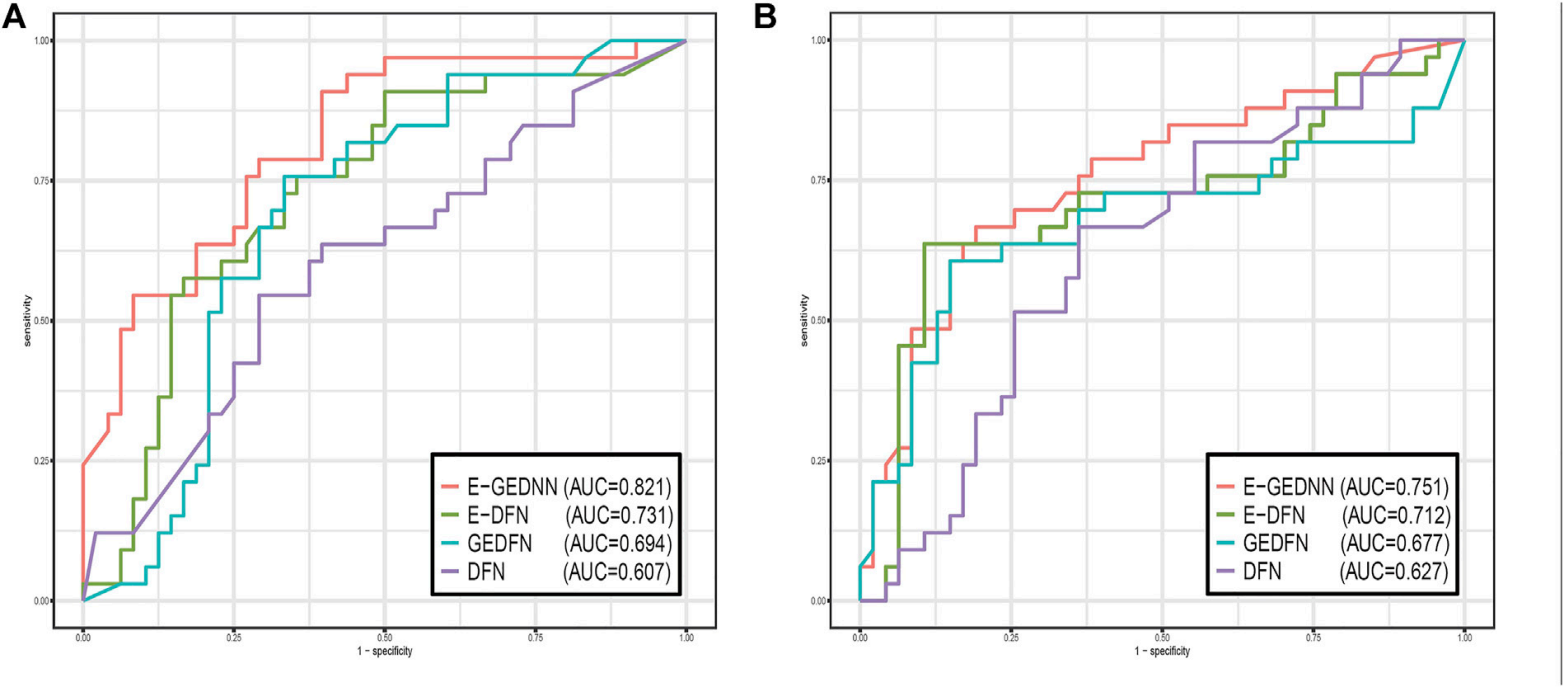
Comparison of the performance of G-EDNN with DNN, E-DNN, GEDFN, and other methods using receiver operating characteristics curves. **(A)** shows the results for GSE28127, and **(B)** shows the results for GSE95496. The legend contains the AUC values of each method on the data sets. Our method has the highest AUC value, indicating that our method outperforms the other methods.

Two data sets, GSE28127 and GSE95496, were used to compare the method of this paper with the above three methods, and as it is a dichotomous problem, we could illustrate the comparison with AUC values, as shown in Figure 2.

From the results of Figure 2, we can see that this method performed better in both data sets compared to other methods. The results of the G-EDNN method are significantly better than those of the GEDFN method, which indicates that intra-omics correlations should be integrated into the analysis process and incorporated into the DNN to better align with biological reality. The results of G-EDNN are better than those of the E-DNN method, primarily owing to the addition of filtering the correlation between SNPs and genes. This indicates that considering the relationship between SNPs and genes at the same time as the foundational consideration of intergenic correlations is more reflective of the actual biological system.

Both the E-DNN method and GEDFN method showed advantages over the general DNN, which indicates that incorporating either SNP–gene association or gene network data can both effectively reduce feature dimensionality and improve prediction accuracy.

Applying all four methods on the two data sets, we found that the results of the first data set are significantly better than the results of the second data set, mainly because of the large difference in SNP numbers between the two data sets. The number of SNPs in the first data set after preprocessing was 2065, while the number of SNPs in the second data set was 6,426. The sample sizes of the two data sets were 501 and 508, respectively. Thus, in the situation of nearly identical sample sizes, the smaller the number of filtered features, the better the results.

### Sample sizes analysis

In genotype–phenotype association analysis and studies, the sample size often has a certain degree of influence on results. For example, for the NETAM method (Lee et al., 2016), its performance was significantly better (larger area under the curve) when the sample size N > 200, while the GSPLS method (Guo et al., 2021) required a sample size of only a few dozen.

**FIGURE 3:**
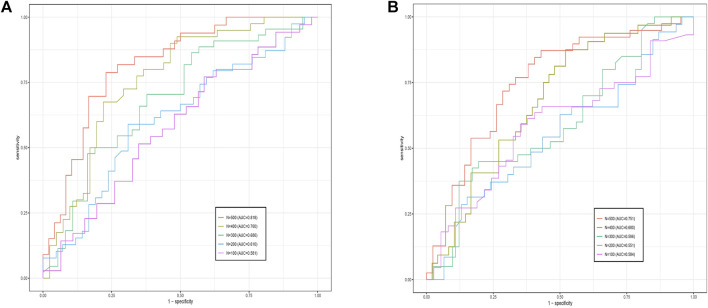
Comparative receiver operating characteristics curves of G-EDNN performance for different sample sizes. The left side shows the results for GSE28127, and the right side shows the results for GSE95496. The legend depicts the sample size and the AUC value for each sample size on the data. As the sample size decreases, corresponding AUC values also decrease.

To validate the effect of sample size on the present method, we randomly selected sample sizes N = 500, 400, 300, 200, and 100 from the data set, and ensured that positive and control samples were in the same proportion during the sample size extraction process. Experiments with both data sets (GSE28127 and GSE95496) showed that the overall trend of AUC values decreased with decreasing sample size, as shown in Figure 3. Comparing the experimental results of the two data sets, we found that the experimental effect of GSE28127 was better than that of GSE95496 with the same sample size; the primary reason, as stated in the prediction analysis, is related to the number of features. It was also found that when data set GSE28127s sample size was less than 200, and data set GSE95496s sample size was less than 300, adding various methods to address the “overfit” problem (e.g., adding a dropout layer, reducing the learning rate, and using other optimizers) was unable to prevent overfit. This means that the sample size that generates an overfitting phenomenon varies by the number of features in the network. Therefore, with the sample sizes being equal, the overfitting phenomenon is more likely to occur for data with a larger number of features.

### Pathway analysis

In contrast to other methods, this method can analyze the pathway relationships between SNP, gene, and phenotype. The G-EDNN method can obtain the weight relationship between each SNP, related genes, and the disease connection. These two layers of weights are multiplied together to obtain the pathway weights (if no edge exists, the edge weights are set to 0), which we name the pathway score (PS) to reflect its importance.

Taking data set GSE95496 as an example, we ranked the PS of the associations between each -ome layer and isolated the top 20 strongest pathways for comparison with acute myeloid leukemia related SNPs and genes in the PhenoScanner database. (Staley et al., 2016). The PhenoScanner database contains 137 genotype–phenotype association data sets, the NHGRI-EBI GWAS catalog, and NHLBI GRASP and dbGaP correlation catalogs. During this comparison process, we did not normalize the weights, mainly because the PS is a two-tier weight product, and normalization would affect the relative proportion of the two weights in the overall PS.

Matching our results with the PhenoScanner database could ascertain whether this paper’s proposed method could identify confirmed key loci or genes. As seen in Table 1, SNPs or genes in six of the top 10 pathways obtained by our sorting appeared in the PhenoScanner database. Among the top 20 pathways obtained by sequencing, nine of the SNPs or genes in the pathway appeared in the PhenoScanner library.

**TABLE 1:** PS values of the top 20 pathways.

	SNP	Gene	PS	Presence in PhenoScanner
1	rs6564261	CFDP1	383.11	Yes
2	rs11915851	ITIH3	341.02	No
3	rs17304995	RFT1	302.16	Yes
4	rs35671032	PRKCD	274.37	No
5	rs1178032	CENPB	270.12	No
6	rs59895335	PRKCE	259.24	Yes
7	rs10781976	BCAR1	257.39	Yes
8	rs113487987	DPYD	252.41	Yes
9	rs76214357	ITIH1	250.26	Yes
10	rs8100824	LRRC25	243.75	No
11	rs116793674	MYL7	243.05	No
12	rs12652555	ERAP1	238.23	No
13	rs56063308	MAP3K7	238.05	Yes
14	rs217361	TMED4	237.97	No
15	rs118052674	CENPB	225.43	No
16	rs72697033	RFX3	223.58	Yes
17	rs1041608	WDR5	221.85	No
18	rs117104394	NISCH	220.17	No
19	rs1471483	MMRN1	220.02	No
20	rs117259301	VPS16	218.36	Yes

## Conclusion

This paper’s proposed G-EDNN model effectively generates a reduced-dimension representation of input data by ensuring smaller numbers of intermediate layer nodes than the number of input values. Our implementation uses SNP data as input to formulate a function that identifies correct gene associations from values computed by the intermediate layer nodes. We tested various architectures of such networks, including structures obtained from different SNP–gene correlation data and intergene correlation data. As our results show, the learned network captured several biologically important pathways within the data. We classified SNP data using the values produced by the neural network’s intermediate layers and compared these values with various other unsupervised methods as well as previous methods of GWAS classification. We conclude that integrating SNP–gene and gene–gene correlation data improves classification performance.

## Discussion and future work

In the process of establishing SNP–gene relationships, we use not only eQTL data, but also the positional relationships between SNP and the gene. Specifically, if the position of the SNP is in the same delimited area as the gene’s position, we believe that there may be an association between that SNP and that gene. There are two main reasons for this approach. First, SNP data are extremely large and plentiful, while the amount of eQTL data containing SNP data is relatively small; considering only eQTL data may lead to sparse results and potentially miss undiscovered relationships. Second, we use both SNP–gene relationships and the gene’s own network data for filtering; without eQTL data mapped to specific gene correlations, analysis may miss certain gene clusters.

In terms of the model’s generalizability, other models are often tested on simulated data, but this paper’s model was not, and while SNP, gene, eQTL and PPI data networks are all correlated, the potential relationships between them were not reflected in simulated data. For example, when we simulated SNP and gene data, eQTL data could not be generated randomly because the related genes in the network might affect their counterpart eQTL data. Therefore, although we initially intended to use simulated data to reflect our method’s generalizability, owing to the above issue, only real data were used to validate our method.

As this method involves setting multiple hyperparameters, the results are sensitive to individual parameters (e.g., hyperparameter α in the loss function); thus, the resultant AUC still has much room for improvement. Therefore, we believe this method may provide a useful foundation for future studies to incorporate inter-phenotype correlations relationships, such as adding prior data on known disease pathways and associations between diseases to analyze potential relationships between known diseases and other diseases.

## Data Availability

The method is available at https://github.com/2017100647/G-EDNN. Publicly available datasets were analyzed in this study. This data can be found here: https://www.ncbi.nlm.nih.gov/geo/query/acc.cgi; http://www.pickle.gr/; https://gtexportal.org/home/datasets.

## Abbreviations

G-EDNN, graph-embedded deep neural network; GWAS, Genome-wide association studies; SNPs: single nucleotide polymorphisms; PPI, protein–protein interaction; eQTL, expression quantitative trait loci; GEO, Gene Expression Omnibus; MAF, minor allele frequency; ROC, receiver operating characteristic; PS, Pathway Score.
